# Crohn’s and Colitis Canada’s 2021 Impact of COVID-19 and Inflammatory Bowel Disease in Canada: Health Care Delivery During the Pandemic and the Future Model of Inflammatory Bowel Disease Care

**DOI:** 10.1093/jcag/gwab034

**Published:** 2021-10-26

**Authors:** Jennifer L Jones, Eric I Benchimol, Charles N Bernstein, James Guoxian Huang, John K Marshall, Mariam S Mukhtar, Sanjay K Murthy, Geoffrey C Nguyen, Gilaad G Kaplan, M Ellen Kuenzig, Parul Tandon, Laura E Targownik, Joseph W Windsor, Alain Bitton

**Affiliations:** 1 Department of Medicine, Dalhousie University, Halifax, Nova Scotia, Canada; 2 SickKids Inflammatory Bowel Disease Centre, Division of Gastroenterology, Hepatology and Nutrition, The Hospital for Sick Children, Toronto, Ontario, Canada; 3 Child Health Evaluative Sciences, SickKids Research Institute, Toronto, Ontario, Canada; 4 ICES, Toronto, Ontario, Canada; 5 Department of Paediatrics and Institute of Health Policy, Management and Evaluation, University of Toronto, Toronto, Ontario, Canada; 6 Department of Internal Medicine, Max Rady College of Medicine, Rady Faculty of Health Sciences, University of Manitoba, Winnipeg, Manitoba, Canada; 7 University of Manitoba IBD Clinical and Research Centre, Winnipeg, Manitoba, Canada; 8 Department of Medicine and Farncombe Family Digestive Health Research Institute, McMaster University, Hamilton, Ontario, Canada; 9 Department of Internal Medicine, King Abdulaziz University Hospital, Jeddah, Saudi Arabia; 10 The Ottawa Hospital IBD Centre, Department of Medicine, University of Ottawa, Ottawa, Ontario, Canada; 11 Mount Sinai Hospital Inflammatory Bowel Disease Centre, University of Toronto, Toronto, Ontario, Canada; 12 Department of Medicine, University of Calgary, Calgary, Alberta, Canada; 13 Department of Community Health Sciences, University of Calgary, Calgary, Alberta, Canada; 14 Division of Gastroenterology and Hepatology, Mount Sinai Hospital, University of Toronto, Toronto, Ontario, Canada; 15 Department of Medicine, McGill University Health Centre, McGill University, Montreal, Quebec, Canada

**Keywords:** COVID-19, Health care delivery, IBD, Telemedicine

## Abstract

The SARS-CoV-2 pandemic has had a profound impact on inflammatory bowel disease (IBD) health care delivery. The implementation of necessary public health restrictions has restricted access to medications, procedures and surgeries throughout the pandemic, catalyzing widespread change in how IBD care is delivered. Rapid large-scale implementation of virtual care modalities has been shown to be feasible and acceptable for the majority of individuals with IBD and health care providers. The SARS-CoV-2 pandemic has exacerbated pre-existing barriers to accessing high-quality, multidisciplinary IBD care that addresses health care needs holistically. Continued implementation and evaluation of both synchronous and asynchronous eHealthcare modalities are required now and in the future in order to determine how best to incorporate these modalities into patient-centred, collaborative care models. Resources must be dedicated to studies that evaluate the feasibility, acceptability and effectiveness of eHealth-enhanced models of IBD care to improve efficiency and cost-effectiveness, while increasing quality of life for persons living with IBD. Crohn’s and Colitis Canada will continue to play a major leadership role in advocating for the health care delivery models that improve the quality of life for persons living with IBD.

**Key Messages**

- The SARS-CoV-2 pandemic has negatively impacted access to IBD care globally.
- The global IBD community has successfully pivoted to alternate models of care, inclusive of virtual care and remote monitoring of disease activity, which will influence health care delivery models beyond the SARS-CoV-2 pandemic.
- Ongoing support for rigorous, patient-oriented research that seeks to understand how best to implement and sustain new models of health care delivery is needed now and in the future.

## INTRODUCTION

The SARS-CoV-2 pandemic has led to a dramatic shift in access to and delivery of care for Canadians living with inflammatory bowel disease (IBD) (1). The implementation of significant public health restrictions globally resulted in the need to physically distance, adopt proper hand hygiene and wear face masks and, in certain settings, personal protective equipment. Combined with the need to conserve health system capacity, these measures have changed how health care is being delivered in both the inpatient and ambulatory care spaces. This change has spurred rapid and widespread implementation of innovative health care delivery models that may not have been possible in the pre-pandemic era (2). These dramatic shifts, supported by a collective sense of urgency, motivation, and shared objectives across government and health care sectors, have provided valuable insights and lessons related to how IBD care is delivered and what clinicians and individuals with IBD value in health care services delivery. These lessons will have an enduring influence on models of IBD care delivery beyond the SARS-CoV-2 pandemic.

## CHANGES IN HEALTH CARE DELIVERY

The SARS-CoV-2 pandemic has had a dramatic impact on the delivery of health care services both for persons living with and without IBD. Most outpatient care has shifted to virtual care, with elective and semi-elective endoscopy and bowel resection surgery volumes dramatically decreased globally at most centres during the pandemic (3). In most cases, these reductions were due to government and institutional policy decisions either to minimize potential exposure to SARS-CoV-2 (especially at the start of the pandemic when personal protective equipment was limited) or to facilitate redeployment of staff to other essential services, such as intensive care units, emergency departments and medical wards.

### Outpatient Visits Replaced by Virtual Care

Perhaps one of the most impressive, rapidly adopted and far-reaching changes in how IBD care has been delivered during the SARS-CoV-2 pandemic relates to a wholesale adoption of eHealthcare delivery options to reduce the risk of transmission of SARS-CoV-2 associated with in-person clinical visits. eHealth includes the use of both synchronous (telephone consults or follow-up visits, video visits) as well as asynchronous (text messaging, eConsultation, health care delivery via social media) virtual delivery health care modalities (4). The convergence of government and health care system information technology infrastructure support (financial support, fast tracking privacy and security threat assessments, reduction in innovation resistance), patient and provider acceptance, and rapid adoption as a result of the mandatory restrictions imposed during the pandemic led to unprecedented health system change on a global scale.

Gastroenterologists around the world have reported dramatic reductions in the use of in-person clinics (4–8). The literature has overwhelmingly demonstrated that providers and persons living with IBD feel that virtual care is an acceptable, feasible and safe method of health care delivery (9). Evaluations have demonstrated consistently high levels of satisfaction among persons living with IBD (10–13). In fact, several studies have observed that the majority of individuals surveyed would prefer virtual visits versus in-person clinics, particularly for matters of routine follow-up (11, 14). In a survey study of 115 individuals with IBD in Italy (of whom 100 completed the questionnaire), trust in telemedicine services for consultation and desire to continue to use telemedicine services was >90% (15). Additionally, in a Portuguese survey study of 973 individuals with IBD, 88.8% supported remote consultations and 77.3% were satisfied with this type of appointment (16). Although less commonly reported, some centres even utilized social media and alternate communication platforms (e.g., WeChat) in order to facilitate the delivery of virtual care (8). Mastronardi et al. conducted a single-centre study in Castella Grotte, Italy between March and April 2020 (17). They allocated 1038 persons with IBD that they were previously followed into two groups: (i) 421 individuals who they continued to assess in-person (chosen because they required biologic transfusions) and (ii) 617 individuals who received telemonitoring (chosen because they were able to self-administer their therapy [oral or subcutaneous therapies: 5-aminosalicylic acid, steroids, thiopurines]). Telemonitoring was scheduled every 3 days, either by remote video call or telephone. They observed significantly more clinical relapses, based on Mayo Score or Harvey-Bradshaw Index, in the telemonitoring group (23.5%) compared to the in-person control group (18.3%). However, there were no significant differences observed in quality of life (assessed using the IBD32Q) between the groups. Since clinical disease activity is poorly correlated with endoscopic disease activity, these data should be interpreted with caution. Additionally, those being seen in person may have a greater disease burden since they were on biologic therapy. Therefore, a comparable relapse rate between the groups suggests that in-person may be preferred for certain patient groups. However, some individuals remain concerned about missed findings without a physical examination and may prefer in-person care (17).

### Endoscopy and Disease Monitoring

Monitoring IBD disease activity during the pandemic has been challenging with the rapid reduction in the number of colonoscopies being performed at medical centres globally. The Netherlands reported a 14.7% net decrease in endoscopy over the course of the pandemic, with some centres reporting much higher decreases (18); this has forced many IBD centres to be creative in their approach to monitoring disease activity. In an international survey, 52 gastroenterologists from 33 countries reported using blood testing, fecal calprotectin (FC) and cross-sectional imaging when endoscopy was not available (5). However, access to cross-sectional imaging was limited in some countries (e.g., Brazil, Canada, and the United States). Even as we begin to emerge from the third wave of the pandemic, it is highly likely that access to cross-sectional imaging will become even more limited due to the cumulative backlog of tests. Although some Canadian centres had no barriers to accessing FC, some centres identified limited access and funding for FC, including the use of at home FC kits. All Canadian centres had access to standard blood testing, and most had usual access to basic diagnostic imaging (e.g., x-rays). The Endoscopic Healing Index is a serum test composed of 13 protein biomarkers to produce a quantitative score ranging from 0 to 100, which was validated against colonoscopy in individuals with Crohn’s disease and may provide another metric for disease monitoring (19).

### Hospital and Surgical Care

In some COVID-19 hot spots, hospitals have come under considerable strain as persons who contract COVID-19 fill hospital beds and increase overall hospital admission volumes. For example, at the height of the pandemic in New York City, individuals with COVID-19 occupied more than 50% of acute care beds (20). In many areas with lower COVID-19 rates, however, the increase in number of hospitalizations for COVID-19 has been offset by reductions on non-COVID-19 hospitalizations, often resulting in overall reductions in hospitalization burden as many individuals elect to administer self-care at home so as to avoid potential exposure. The rates of ICU admission have increased in most regions as a result of COVID-19, particularly in hot spots. As critical illness cannot be deferred, COVID-19 has compounded general ICU admissions, leading many centres to develop ancillary ICU beds in other parts of the hospital. Some regions, such as Italy and India, have had to implement rationing of ICU beds, ventilators and oxygen due to overwhelming demands at pandemic peaks.

Similar to trends in the general population, hospitalizations for IBD declined following the onset of the pandemic (10, 21). In Madrid, Spain, the rate of IBD-related hospitalizations and visits to the emergency departments decreased by 50% and 58%, respectively, compared with rates in the same period the previous year (10). In a nationwide Dutch study, combined IBD-related endoscopic and surgical services decreased by 59.7% at the national peak of the pandemic in April 2020 as compared to pre-pandemic rates in the preceding year. Over the duration of the pandemic, endoscopic and surgical procedures showed a net decrease of 14.7% (1443 procedures) and 5.5% (33 procedures), respectively (18). In Alberta, 5.7% of patients reported a delay of surgery by a median of 10 weeks (22).

The inevitable hurdle that will be faced by practitioners and institutions once elective services resume to normal capacity will be managing the backlog of cases that have accumulated, most of which would likely still have an active indication for intervention, and which may have become more urgent due to delays in investigation and treatment.

### Multidisciplinary Care

Complex medical, surgical and psychosocial issues associated with IBD often require specialized expertise from a multidisciplinary team that includes gastroenterologists, pharmacists, surgeons, psychologists and dietitians (23, 24). This type of multidisciplinary care delivery is often restricted to large academic centres (24–26). An integrated model of care in IBD has the potential to improve the quality of care, patient satisfaction, and mental and physical health and to reduce health care costs (23, 27, 28). During the pandemic, multidisciplinary visits have often been continued virtually with the individual seen on same day by different members of the multidisciplinary team in order to make joint treatment decisions (29, 30).

Before the SARS-CoV-2 pandemic began, it was recognized that IBD specialist nurses, the main point of access for persons with IBD, played a pivotal role in providing education about disease, medications, monitoring of therapy, basic dietary advice, within-scope psychological and emotional support, access to service and telephone advice, particularly during periods of disease flare (25, 31, 32). The SARS-CoV-2 pandemic has led to a significant increase in the reliance on IBD nurses to provide essential services to persons with IBD remotely. Both synchronous and asynchronous virtual care through telephone and e-mail have been provided by IBD nurses as an important communication pathway for people with IBD needing access to specialist support and advice and facilitating avoidance of clinic appointments and hospital admission (24, 33, 34). IBD nurses have also played an important role in the identification of high levels of distress (already prevalent in IBD and exacerbated by the pandemic) through their remote interactions with patients and have been able to offer virtual modes of psychologic support. Digital eHealth systems have enabled screening of psychologic well-being and delivery of solutions such as app-based, cognitive–behavioral therapy (9). For practices without access to an IBD nurse, the aforementioned responsibilities typically fell on the gastroenterologist or other physicians who, in addition to maintaining an acceptable standard of care for those with IBD, had to ensure safe work practices for themselves, staff and those still required to attend clinic in person; provide general COVID-19 and IBD advice; advise on the safety of IBD medications and COVID-19 vaccines; and identify when it would be necessary to refer an individual for outside specialist help (e.g., dieticians or mental health professionals). Once again, virtual care was received as a tremendous benefit in being able to accommodate this additional workload.

## MEDICAL THERAPY DURING THE PANDEMIC

### Immunomodulatory Therapy and Risk of Severe COVID-19

At the beginning of the pandemic, there was concern among people with IBD that being on immunosuppressive medications might enhance the risk for either acquiring SARS-CoV-2 infection or having more severe COVID-19. Hence, there was concern for individuals with IBD either not being adherent with medications or not accessing them at appropriate intervals due to fear about attending infusion centres or even going into public pharmacies to fill prescriptions. Early assurances from the SECURE-IBD registry regarding the use of anti-tumour necrosis factors helped to reduce anxiety related to IBD biologic use, but concerns remain for other therapies (e.g., steroids). The risk of severe COVID-19 across medication groups are reviewed in detail in the article on ‘Clinical risk factors and IBD medications’ (35). The IBD population was advised early in the pandemic not to interrupt their medication, as this may lead to disease relapse, which could necessitate a new prescription of corticosteroids or hospitalization. These events were considered to pose a greater risk than the medications used to treat IBD (36).

### Provider Prescribing and Treatment Adherence

While data were emerging from the SECURE-IBD registry and experts were providing guidance on medication usage during the pandemic, little information relating to what individuals with IBD and their providers actually did with IBD-specific medications during the pandemic exists. In a Vancouver, BC general gastroenterology clinic, a retrospective chart review of 241 people with IBD compared medication adherence rates after telehealth visits (*n* = 113 individuals) with in-person visits (*n* = 128 individual). The majority of individuals had IBD as their primary gastrointestinal problem and two-thirds were using biologic therapy. Prescription fill rates for patients seen through telehealth (98.2%) were higher than in-person visits (89.1%, *P* = 0.004). Excluding biologic therapies, the prescription fill rate was 94.7% in telehealth group and 81.4% in in-person group (odds ratio: 4.11; 95% confidence interval: 0.88, 19.27). Reasons for this difference were not clear but may include the process of getting the prescriptions to the pharmacies (i.e., electronically faxed directly to pharmacies versus individuals being handed a prescription during the in-person visit forcing another visit to a public establishment [the pharmacy]) or systematic differences between those people seen in person versus those seen via telehealth, which may influence medication adherence. In a study out of Denmark, 14.3% of 400 participants using IBD medication paused or stopped their IBD treatment during the initial phase of the pandemic. The majority (61.4%) discontinued IBD medications either due to remission or because of side-effects (37); however, corticosteroids were the most frequently discontinued medication, and it is not clear if discontinuation was due to completion of a short-course of steroids or in response to risks associated with SARS-CoV-2 infection. Only five respondents stated that the pandemic contributed to their decision to discontinue therapy.

### Access to Biologic Therapies

While adherence to medications may not have changed significantly, there were concerns that public health policies might limit access to biologic therapies due to a reduction in infusion capacity or closure of infusion clinics. In a study of 398 participants attending University of Alberta or University of Calgary IBD clinics from March 2020 through July 2020, only 2.2% reported a delay in biologic infusions for a median of 2 weeks; it is unknown how representative this is of other clinic models across Canada (22). As a result of concerns about attending health clinics for infusions at two hospitals in the United Kingdom in March 2020, a program was implemented in which all hospital-based intravenous infusions of infliximab were switched to home-based, self-administered, subcutaneous injections of CT-P13, an infliximab biosimilar, with all but 3 of 163 participants tolerating the switch without early flares or intolerance (38).

## IBD CARE DELIVERY: LESSONS FOR THE FUTURE

The rapid and transformative health care delivery changes that have been made and lessons learned throughout the SARS-CoV-2 pandemic to date have facilitated fast-paced innovation that has leveraged and built upon previous eHealth implementation success in the IBD space. These innovations will need to continue well beyond the current pandemic in order to improve exacerbations in access to quality care for persons living with IBD.

Another area of health care delivery in the ambulatory IBD space that will be transformed in the future relates to remote monitoring of clinical factors proven to impact disease-related outcomes. Remote monitoring of patient-reported outcomes, disease activity, medication adherence, nutritional status, mental health and well-being through the use of digital health technology will facilitate implementation of a more comprehensive and holistic care model; one that has historically been difficult, if not impossible, to implement through traditional clinic-based models of care and with limited IT infrastructure. These models will also serve to engage and empower many people living with IBD with respect to self-management and autonomy. Apps on mobile devices or web-based programs through which individuals can report symptoms to providers who then respond with a timely intervention have the potential to improve health outcomes. Only a few randomized trials have evaluated such digital health technology and its potential impact on health outcomes. So far, improved disease-specific quality of life and reduced health care utilization including outpatient visits and hospitalizations have been reported (39–41).

The future model of IBD care will almost certainly include a hybrid of in-person and virtual care delivery strategies, supported by synchronous and asynchronous eHealth technology. These eHealth platforms will be integrated within the electronic medical record along with clinical data (i.e., symptoms, laboratory and biomarker data) derived from both active and passive (e.g., smart watches) digital remote monitoring tools (42). Given the exponential rise in the routine collection of clinical data through remote monitoring activities, artificial intelligence (AI) algorithms based on evidence-based care pathways will be needed in order to gather, classify and interpret data, as well as support clinicians (i.e., clinical decision supports through embedded smart algorithms). These eHealth and digital monitoring tools will ideally be applied within the context of a multidisciplinary care setting in which the ability to respond to the data and to adequately address individual needs will be facilitated. How these technologies are designed and implemented within clinical care settings as well as the individuals’ lives will need to be balanced by a patient-centred approach, informed by extensive stakeholder engagement.

Much work in relation to health care delivery advocacy and policy is needed now and into the future. Pre-existing barriers in access to high-quality, multidisciplinary care have been exacerbated by the SARS-CoV-2 pandemic. National guidelines for the use and implementation of virtual care, digital remote monitoring and the application of AI will need to be developed and standardized, while taking into account the unique environmental considerations of each province, institution and clinic. As technology evolves, integration of all eHealth platforms within an electronic medical record will allow for more seamless tracking of data and, therefore, a greater likelihood of technology adoption.

Several initiatives that were being developed in the Canadian landscape prior to the pandemic have evolved and will play an important role in the future of IBD care (Table 1). Developed by Crohn’s and Colitis Canada, the Promoting Access and Care through Centres of Excellence (PACE) Telemedicine Program in Ontario, Canada has already demonstrated that use of video consultation for geographic areas with minimal access to specialized IBD care was feasible, reduced wait times and was cost-saving (43). These benefits were realized while maintaining high-value care. Teleconsultation through the PACE program has been successful in Ontario and will be expanded across Canada in the near future. MyGut, a mobile app inaugurated in 2020, is a platform for health care provider–patient interaction, remote monitoring, and tele-education. Also being implemented is the IBD Global Rating Scale, a web-based, periodic self-reporting system for IBD care providers to help improve and standardize the quality of IBD care across Canada. The Crohn’s and Colitis Canada COVID-19 IBD Taskforce should remain as a general IBD knowledge translation channel even after the pandemic.

**Table 1.** Post-pandemic care in IBD: Canadian initiatives

Activity	Current	Future
Teleconsultation	PACE Telemedicine Program	Expansion across Canada
Telemonitoring	MyGut mobile device application	MyGut implementation
IBD quality of care	Guidelines, IBD GRS, PACE IBD care pathways	Implementation of IBD GRS and PACE IBD care pathways
Knowledge translation	CCC COVID-19 and IBD Taskforce	Dedicated long-term knowledge translation committee

CCC, Crohn’s and Colitis Canada; GRS, Global Rating Scale; IBD, Inflammatory bowel disease; PACE, Promoting Access and Care through Centres of Excellence.

An increasing amount of health research utilizes patient partners as invaluable resources in defining the research questions, participating in literature reviews and providing stakeholder assessments and feedback. As the new models of care first introduced during the pandemic continue to evolve in the post-pandemic era, they must do so with users at top of mind. Specifically, people with IBD need to be partners in the design of the post-pandemic multidisciplinary care models, including synchronous and asynchronous care, data tracking (e.g., wearable technology or mobile apps), and in training and testing AI algorithms for care pathways. Individual preferences should also be incorporated in the type and location of care provided. A critical component of this patient-centred approach is integrated knowledge translation strategies where end-stage users are included in the research.

The COVID-19 pandemic is a global crisis that will leave its imprint for years to come and will reshape the way medicine is practised. The medical community has had to respond, adapt and effect change rapidly. This change will pave the way for a transformed and enhanced model of IBD care and will bring about reflection on our readiness for future challenges of such magnitude as the COVID-19 pandemic. Rapidly emerging literature in relation to the widespread implementation of virtual care modalities in combination with the use of non-invasive disease monitoring strategies have demonstrated the acceptability, feasibility and safety of these approaches during the pandemic.
